# Reciprocal Inhibition of Adiponectin and Innate Lung Immune Responses to Chitin and *Aspergillus fumigatus*

**DOI:** 10.3389/fimmu.2019.01057

**Published:** 2019-05-10

**Authors:** Nansalmaa Amarsaikhan, Dylan J. Stolz, Amber Wilcox, Ethan M. Sands, Angar Tsoggerel, Haley Gravely, Steven P. Templeton

**Affiliations:** Department of Microbiology and Immunology, Indiana University School of Medicine—Terre Haute, Terre Haute, IN, United States

**Keywords:** adiponectin, chitin, *Aspergillus fumigatus*, lung immune responses, inflammatory cytokines, eosinophils, neutrophils

## Abstract

Chitin is a structural biopolymer found in numerous organisms, including pathogenic fungi, and recognized as an immune-stimulating pathogen associated molecular pattern by pattern recognition molecules of the host immune system. However, programming and regulation of lung innate immunity to chitin inhalation in the context of inhalation of fungal pathogens such as *Aspergillus fumigatus* is complex and our understanding incomplete. Here we report that the systemic metabolism-regulating cytokine adiponectin is decreased in the lungs and serum of mice after chitin inhalation, with a concomitant decrease in surface expression of the adiponectin receptor AdipoR1 on lung leukocytes. Constitutive lung expression of acidic mammalian chitinase resulted in decreased inflammatory cytokine gene expression and neutrophil recruitment, but did not significantly affect lung adiponectin transcription. Exogenous recombinant adiponectin specifically dampened airway chitin-mediated eosinophil recruitment, while adiponectin deficiency resulted in increased airway eosinophils. The presence of adiponectin also resulted in decreased CCL11-mediated migration of bone marrow-derived eosinophils. In contrast to purified chitin, aspiration of viable conidia from the high chitin-expressing *A. fumigatus* isolate Af5517 resulted in increased neutrophil recruitment and inflammatory cytokine gene expression in adiponectin-deficient mice, while no significant changes were observed in response to the isolate Af293. Our results identify a novel role for the adiponectin pathway in inhibition of lung inflammatory responses to chitin and *A. fumigatus* inhalation.

## Introduction

Chitin is a ubiquitous biopolymer of high-tensile strength found in many eukaryotes, including fungi, crustaceans, arthropods, and helminths (1–3). In contrast, chitin synthesis is absent in mammalian tissues, where chitin-degrading chitinases are widely expressed and highly conserved (4). Defects in chitinase-mediated chitin degradation are associated with airway accumulation of chitin, inflammatory cells, and development of pulmonary fibrosis (5). Pattern recognition of chitin particles by an array of other microbial ligand-binding receptors drives immune responses that vary based on chitin particle size and acetylation, ranging from inflammatory to regulatory profiles (2, 6, 7). In addition, inflammatory cytokine expression in response to purified or fungal chitin exposure includes secretion of IL-1, IL-4, IL-13, IL-10, and IL-17 (6, 8, 9). However, programming and regulation of lung innate immunity to chitin inhalation in the context of inhalation of fungal pathogens such as *Aspergillus fumigatus* is complex and our understanding incomplete.

The adipose tissue cytokine adiponectin is well-known as a regulator of insulin responsiveness and fatty acid oxidation, yet also exerts anti-inflammatory effects on macrophages, innate lymphocytes, eosinophils, and neutrophils (10). Moreover, adiponectin is constitutively high in the plasma of healthy, lean individuals (11–13), and significantly decreased in association with the chronic inflammatory diseases obesity and asthma (14–16). Adiponectin deficiency resulted in increased lung inflammation and pathology in mouse models of allergic asthma and ozone inhalation, supporting an anti-inflammatory role for adiponectin in lung immune pathology (17, 18). Recently, obese and adiponectin-deficient mice were reported deficient in clearance of the food-borne bacterial pathogen *Listeria monocytogenes* due to increased bone marrow inflammation and defective granulopoiesis (19). Adiponectin inhibits macrophage activation and cytokine secretion in response to bacterial lipopolysaccharide, potentially via blockade or desensitization of inflammatory signaling pathways (10). However, the effect of adiponectin on lung inflammation induced by fungal pattern associated molecular patterns (PAMPs) is unknown.

Herein we report that lung and serum adiponectin were decreased after aspiration of purified chitin or conidia of a high chitin-expressing strain of *A. fumigatus*. Co-aspiration of chitin with recombinant adiponectin or adiponectin deficiency resulted in decreased or increased lung eosinophil recruitment, respectively, while numbers of neutrophils and alveolar macrophages were not affected. Eosinophils, neutrophils, and alveolar macrophages expressed surface AdipoR1 that was decreased upon chitin inhalation, and eosinophil migration in response to CCL11 (eotaxin-1) was decreased in the presence of adiponectin. In contrast to the response to purified chitin and fungal conidia of the strain Af293, aspiration of the high-chitin expressing Af5517 conidia by adiponectin-deficient mice resulted in significantly increased accumulation of lung neutrophils and expression of inflammatory cytokines. Our results identify a novel role for the adiponectin pathway in inhibition of lung inflammatory responses to chitin and *A. fumigatus* inhalation.

## Results

### Lung Inflammatory Responses to Purified Chitin Aspiration Are Associated With Decreased Lung and Serum Adiponectin and Decreased Leukocyte Surface AdipoR1 Expression

**Figure 1:**
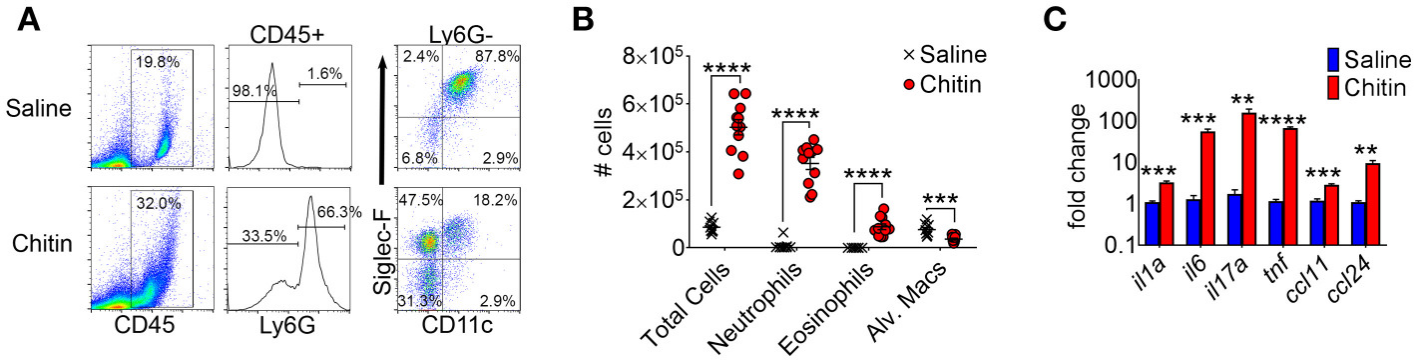
Lung inflammatory responses after chitin aspiration. **(A–C)**, BALB/c mice were given 5 × 10^3^ purified chitin particles by aspiration on consecutive days, with BALF, lung homogenates, and serum harvested for analysis 24 h after the second challenge. **(A)** Representative flow cytometric dot plots of CD45^hi^ cells from control (saline) and chitin-aspirated mice. **(B)** Total number of BALF cells, Ly6G^hi^ neutrophils, Ly6G^lo^SiglecF^hi^CD11c^lo^ eosinophils, and Ly6G^lo^SiglecF^hi^CD11c^hi^ alveolar macrophages isolated from saline or chitin-aspirated mice as determined by flow cytometry. **(C)** Quantitative RT-PCR analysis of RNA from total lung homogenates of chitin-aspirated mice. *N* = 6–10/group. Data are shown as a summary of 2 experiments. Error bars in all figures represent standard error of mean. **p < 0.01. ***p < 0.001. ****p < 0.0001.

A previous study reported lung eosinophil accumulation in response to inhalation of purified chitin particles (20), and since the initial finding chitin-mediated neutrophil recruitment was also described (5). We confirmed that CD45^hi^Ly6G^hi^ neutrophils and CD45^hi^Ly6G^lo^CD11c^lo^SiglecF^hi^ eosinophils were markedly increased in the airways of chitin-aspirated mice, while resident CD45^hi^Ly6G^lo^CD11c^hi^SiglecF^hi^ alveolar macrophages were decreased (Figures 1A,B). In addition, inflammatory cytokines and chemokines that drive recruitment and/or activation of these cells, including IL-1α, IL-6, IL-17A, TNF, CCL11 (eotaxin-1), and CCL24 (eotaxin-2), were also increased relative to control (saline aspirated) mice (Figure 1C), confirming an inflammatory phenotype in the lungs of chitin-aspirated mice.

**Figure 2:**
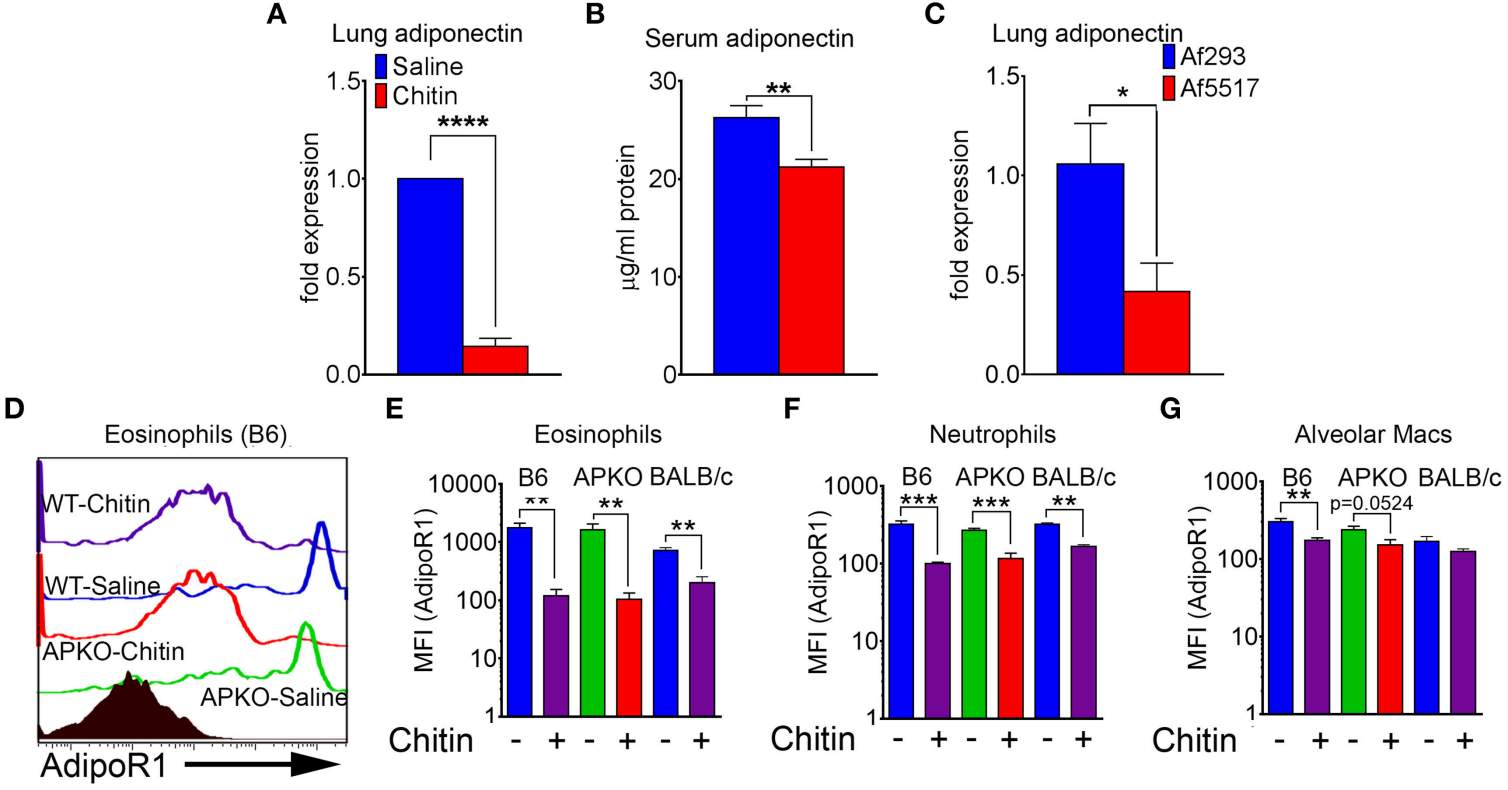
Decreased lung, serum adiponectin and surface leukocyte AdipoR1 after chitin aspiration. **(A)** Fold change in expression of adiponectin mRNA in lung homogenates as determined by quantitative RT-PCR. *N* = 6–10/group. **(B)** Concentration of serum adiponectin protein in saline and chitin-aspirated mice as determined by ELISA. *N* = 5–6/group. **(C)** Fold expression of lung adiponectin gene expression in BALB/c mice that repeatedly aspirated 2 × 10^6^ conidia of low/normal (Af293) or high (Af5517) chitin-expressing isolates. **(D–G)** BALF was harvested and eosinophils were analyzed by flow cytometry for AdipoR1 expression. **(D)** Representative histograms of AdipoR1 staining on eosinophils. **(E–G)** Summary of median fluorescence intensities of AdipoR1 staining on eosinophils, neutrophils, and alveolar macrophages from 2 experiments with wild-type or adiponectin-deficient mice, B6 background, or wild-type mice, BALB/c background. Data are a summary of 2–3 experiments. *N* = 6–10/group. *p < 0.05. **p < 0.01. ***p < 0.001. ****p < 0.0001.

We next compared adiponectin (*adipoq*) transcript levels in lung tissue and human lung epithelial cells, and adiponectin protein levels in the serum of saline and chitin-aspirated mice. Notably, the low baseline level of lung transcription of adiponectin measured in saline-aspirated mice was significantly decreased upon aspiration of chitin particles (Figure 2A and data not shown), with a concomitant decrease in the concentration of serum adiponectin protein (Figure 2B). In contrast, curdlan (β-1,3-glucan) aspiration did not affect lung adiponectin transcription and neither chitin nor curdlan aspiration resulted in significantly altered lung leptin expression (Figure S1). In addition, we did not observe a consistent change in transcription of adiponectin in response to chitin in human lung epithelial (A549) cells stimulated with chitin and/or TNF (Figure S2A). In order to determine if fungal chitin results in decreased relative adiponectin gene expression, we compared gene expression in lungs from mice that repeatedly aspirated conidia of a normal/low chitin-expressing isolate of *A. fumigatus* (Af293) with mice that aspirated a high chitin-expressing isolate (Af5517) (21, 22). Our results indicated significantly lower adiponectin transcription in the lungs of Af5517-challenged mice when compared to repeated Af293 aspiration (Figure 2C). In contrast, purified chitin or swollen/fixed Af5517 conidia incubated with human lung A549 cells was not associated with a significant change in adiponectin expression, while an increase was observed in A549 cells incubated with swollen/fixed Af293 conidia (Figure S2A). Increased *adipoq* expression was associated with a marked increase in IL-6 mRNA and protein in response to Af293 conidia, with a lesser increase in IL-6 in response to Af5517 (Figures S2C,D). Thus, particulate chitin aspiration is associated with increased inflammation and decreased constitutive expression of whole lung mRNA and serum adiponectin protein, although this phenotype was not reflected in human lung epithelial A549 cells.

AdipoR1 is considered a major receptor mediating the anti-inflammatory effect of adiponectin on immune cells (10). We therefore wanted to determine if AdipoR1 was expressed on the surface of lung leukocytes and how this expression was modified by chitin aspiration. The low levels of lung eosinophils isolated from naïve mice expressed the highest relative surface staining of AdipoR1 as measured by flow cytometry, and chitin aspiration was associated with a significant decrease in wild-type B6, BALB/c and adiponectin KO (B6 background) mice (Figures 2D,E). Although surface expression of AdipoR1 was not as high in neutrophils and alveolar macrophages as compared to eosinophils by fluorescence intensity, AdipoR1 staining was also decreased after chitin aspiration in these populations (Figures 2F,G). In A549 cells, *adipoR1* transcription was not significantly modulated in response to chitin or swollen/fixed conidia (Figure S2B). Thus, chitin aspiration may result in inhibited responsiveness to adiponectin by decreased surface AdipoR1 in multiple lung leukocyte populations.

**Figure 3:**
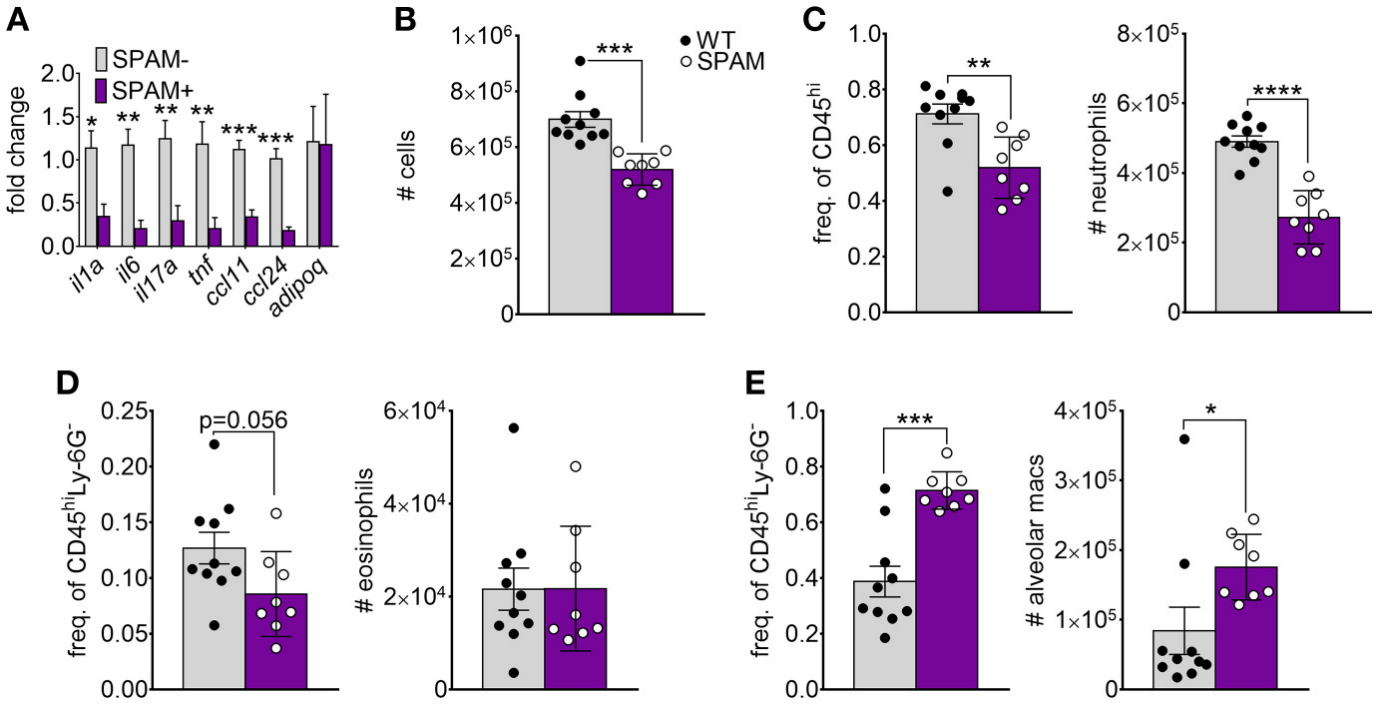
Decreased chitin-induced inflammation with transgenic expression of lung AMCase. SPAM transgenic mice or non-transgenic littermates were given chitin particles by involuntary aspiration as described for Figure 1. **(A)** RNA from lung homogenates was analyzed by qRT-PCR for the indicated cytokines. *N* = 6–10/group. **(B–E)** Total BALF leukocytes **(B)**, frequency (left panels) and total numbers (right panels) of neutrophils **(C)**, eosinophils **(D)**, and alveolar macrophages **(E)** as determined by flow cytometry. Data are a summary of 2 experiments. *p < 0.05, **p < 0.01, *** *p* < 0.001, ****p < 0.0001.

Previous studies showed that transgenic lung airway expression of acidic mammalian chitinase (SPAM) inhibited chitin-mediated recruitment of eosinophils and M2 macrophage activation (20, 22, 23). We wanted to determine if chitin degradation in chitin-aspirated SPAM mice resulted in increased lung adiponectin transcription compared to non-transgenic mice. We observed a consistent decrease in lung mRNA from the genes that encode IL-1a, IL-6, IL-17A, TNF, CCL11, and CCL24 in SPAM+ mice compared to SPAM- mice (Figure 3A). In contrast, adiponectin gene expression was not significantly altered. Total lung cells were decreased in SPAM+ mice (Figure 3B), mainly reflected by decreased neutrophils (Figure 3C). Eosinophils in SPAM+ mice were not significantly decreased (Figure 3D), while alveolar macrophages were increased in transgenic animals (Figure 3E). Although inflammatory responses were significantly inhibited with transgenic expression of lung AMCase, enhanced enzymatic chitin degradation was not associated with a concomitant increase in lung adiponectin gene expression.

### Inhibition of Chitin-Mediated Eosinophil Recruitment by Adiponectin

**Figure 4:**
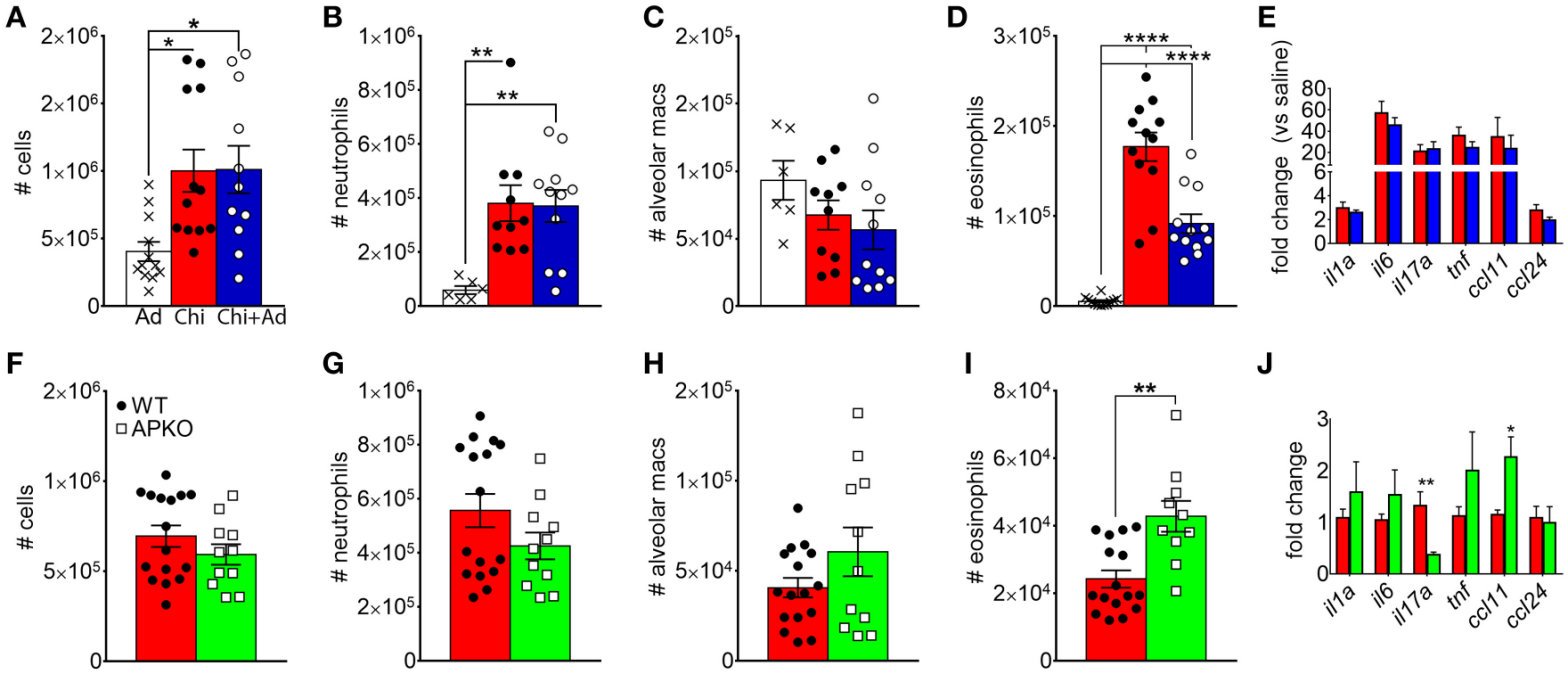
Inhibition of chitin-mediated airway eosinophil recruitment by adiponectin. BALB/c **(A–E)** or C57BL/6 wild-type (WT) or adiponectin-deficient (APKO) **(F–J)** mice aspirated chitin particles as described for Figure 1, with BALF analyzed by flow cytometry 24 h after the second challenge. **(A–E)** Mice aspirated 0.5 μg/Kg recombinant adiponectin alone, chitin particles alone, or particles + adiponectin. **(A,F)** Total CD45hi leukocytes, **(B,G)**, neutrophils. **(C,H)**, alveolar macrophages **(D,I)**, eosinophils. **(E,J)** Quantitative RT-PCR of lung homogenate RNA to determine expression of the indicated cytokines/chemokines. *N* = 6–10/group. Data are a summary of 2–3 experiments. *p < 0.05, **p < 0.01, ****p < 0.0001.

Since chitin aspiration is associated with decreased adiponectin in addition to inducing lung accumulation of eosinophils, we also wanted to determine if adiponectin inhibits chitin-mediated eosinophil and/or neutrophil accumulation. In order to accomplish this, we first co-aspirated BALB/c mice with recombinant murine adiponectin (0.5 μg/Kg) along with chitin particles and compared eosinophil recruitment to mice that aspirated adiponectin or chitin alone. Although numbers of total leukocytes, neutrophils, and alveolar macrophages remained unchanged with adiponectin co-aspiration (Figures 4A–C), airway eosinophils were significantly decreased with co-aspiration compared to mice that only aspirated purified chitin (Figure 4D). Despite this reduction in eosinophils, we did not observe a significant decrease in the transcripts of inflammatory cytokine genes or the eosinophil-attracting chemokines CCL11 and CCL24 (Figure 4E). Reciprocally, when mice deficient in adiponectin aspirated chitin particles, airway eosinophils, but not other leukocytes, were increased in comparison to wild-type C57BL/6 mice (Figures 4F–I), although the relative numbers of airway eosinophils were lower in B6 background mice compared to BALB/c mice (Figure 4D). Lung *ccl11* mRNA was increased in chitin-aspirated, adiponectin-deficient mice, while *il1a, il6, tnf*, and *ccl24* were not significantly altered, and *il17a* mRNA was decreased (Figure 4J). Thus, administration of exogenous adiponectin resulted in a specific decrease in chitin-mediated lung eosinophil accumulation with increased eosinophils in the absence of adiponectin.

**Figure 5:**
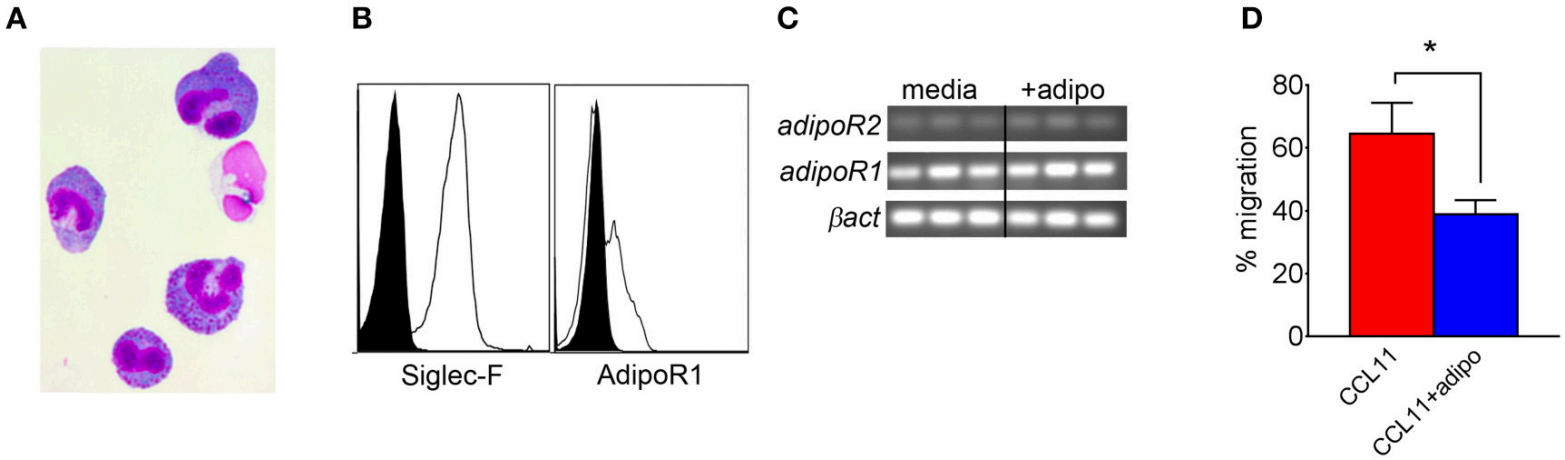
Decreased bone marrow-derived eosinophil migration in response to CCL11 in the presence of adiponectin. Bone marrow-derived eosinophils (BMEs) were characterized by microscopy for morphology **(A)**, flow cytometry for surface expression of Siglec-F and *adipoR1* **(B)**, RT-PCR for transcription of *AdipoR1* and *adipoR2* **(C)**, and with a transwell migration inhibition assay in response to recombinant CCL11 in the presence of absence of recombinant adiponectin, with three replicates **(D)**. Data are representative of two experiments with similar results. *p < 0.05.

We also compared chemotaxis of bone marrow-derived eosinophils (BMEs) in response to CCL11 (eotaxin-1) in the presence or absence of adiponectin, verifying the BME phenotype by microscopy (Figure 5A) and flow cytometry/qRT-PCR for surface expression of Siglec-F and/or AdipoR1/R2 (Figures 5B,C). Pre-incubation with recombinant adiponectin resulted in decreased transwell migration of BMEs in response to CCL11 (Figure 5D). Therefore, our results from multiple approaches suggest that adiponectin inhibits chitin-mediated eosinophil recruitment and migration.

### Fungal Isolate-Specific Increase in Lung Neutrophil Recruitment and Inflammatory Cytokine Expression in Adiponectin-Deficient Mice

**Figure 6:**
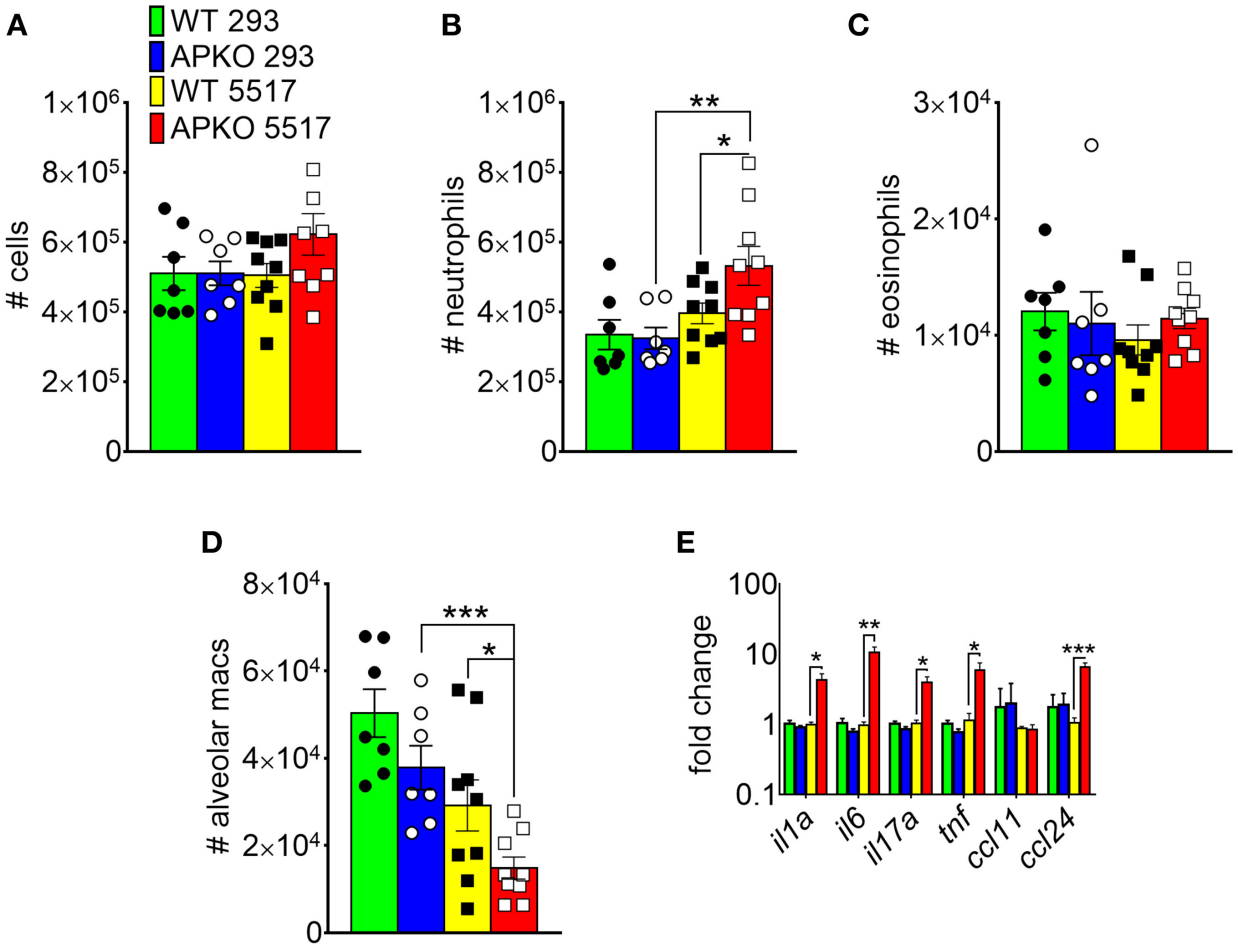
Fungal isolate-specific modulation of airway leukocyte recruitment and increased lung inflammatory cytokine expression in adiponectin-deficient mice in response to *A. fumigatus* aspiration. Wild-type or adiponectin-deficient mice aspirated 5 × 10^6^ conidia from Af293 or Af5517 isolates; lungs were harvested 48 h post-challenge for analysis of BALF cells for the indicated cell types by flow cytometry **(A-D)** or lung homogenate cytokine mRNA by qRT-PCR **(E)**. *N* = 6–10/group. Data are a summary of two independent experiments for each fungal isolate. *p < 0.05, **p < 0.01, ***p < 0.001.

In *A. fumigatus*, chitin is a cell wall structural molecule that is covalently linked with other immune-stimulating molecules like β-1,3-glucan that are co-exposed on the surface of swollen, germinating conidia, and hyphae (24). Thus, innate recognition of chitin in response to fungal inhalation and infection occurs with co-recognition of other fungal PAMPs. We previously reported that immune responses to the high chitin-expressing *A. fumigatus* isolate Af5517 were skewed toward type 2 immune responses with increased eosinophil recruitment (22). Thus, we wanted to compare the response to aspiration of conidia from the Af293 and Af5517 isolates in the absence of adiponectin. In contrast to the unchanged response to the normal/low chitin-expressing Af293 isolate in wild-type and adiponectin deficient mice, aspiration of viable Af5517 conidia resulted in increased neutrophil recruitment with decreased alveolar macrophages compared to wild-type mice in the absence of adiponectin (Figures 6A,B), while eosinophils were not significantly changed in response to either isolate (Figure 6C). Furthermore, alveolar macrophages were decreased in the absence of adiponectin in response to Af5517 (Figure 6D). Consistent with increased inflammation, *il1a, il6, il17a*, and *ccl24* lung mRNA levels were increased in adiponectin KO mice in response to Af5517, but not Af293 conidia (Figure 6E). Our results demonstrate a fungal isolate-specific increase in lung inflammation in adiponectin-deficient mice in response to aspiration of *A. fumigatus* conidia with a distinct profile in comparison to responses to purified chitin.

## Discussion

In this study, our initial goal was to determine if adiponectin inhibited lung chitin-mediated eosinophil recruitment, since others had shown that adiponectin inhibited allergic lung inflammation and eosinophil migration and adhesion (17, 25, 26). For this we used an established model of lung eosinophil recruitment that involves inhalation of purified chitin (Figure 1). We used a commercially prepared purified large chitin particle suspension (<70 μm) due to the established effect on eosinophil recruitment, despite differences in size and acetylation with purified fungal chitin particles (2). In mice that aspirated purified chitin, we observed that expression of lung adiponectin and adiponectin receptor AdipoR1 on eosinophils, neutrophils, and alveolar macrophages were decreased (Figure 2). However, our results with human lung epithelial A549 cells did not display similar changes in adiponectin or AdipoR1 gene expression in response to chitin or fungal conida (Figure S2). Increased constitutive lung chitinase activity in SPAM transgenic mice was sufficient for decreased lung inflammatory cytokines and leukocytes in comparison to non-transgenic littermates, but was not sufficient for increased adiponectin transcription (Figure 3). Mouse aspiration of recombinant adiponectin resulted in a specific decrease in lung chitin-mediated eosinophil recruitment, while lung inflammatory cytokines and chemokines were not significantly modulated (Figures 4A–D). Reciprocally, eosinophils and the corresponding eosinophil chemokine CCL11 (eotaxin-1) were increased in adiponectin-deficient mice compared with wild-type (Figure 4I). The presence of recombinant adiponectin also resulted in decreased bone marrow-derived eosinophil migration in response to CCL11 (Figure 5). Thus, our results from multiple approaches collectively support a role for adiponectin in inhibition of chitin-mediated eosinophil recruitment, and suggest a reciprocal chitin-driven decrease in lung adiponectin and adiponectin receptor expression.

Although our results were consistent with an inhibitory role for adiponectin in eosinophil recruitment and migration in response to purified chitin, adiponectin is also known to inhibit activation and/or cytokine secretion of NK cells, neutrophils, dendritic cells, and γδ T cells (10). Although we did not see decreased recruitment of neutrophils and alveolar macrophages in the presence of adiponectin, AdipoR1 expression on these cells was still relatively decreased after chitin aspiration compared to cells from naïve animals (Figures 2F,G). This suggests that other adiponectin-regulated cellular processes could be decreased by chitin aspiration in these cells. Lung macrophages and γδ T cells may also be activated by particulate chitin inhalation (8, 20). Although we did not observe changes in adiponectin or adipoR1 mRNA in lung epithelial A549 cells in response to chitin or fungal conidia (Figure S2), other groups have also reported AdipoR1 expression in these cells as well as inhibition of cell proliferation and viability in the presence of adiponectin (27–30). Thus, macrophages, γδ T cells, and lung epithelial cells are interesting targets for future studies to determine cell-specific modulation of fungal responses by adiponectin.

Aspiration of the fungal conidia isolate Af5517, which expresses increased chitin covalently linked with other immune-stimulating fungal PAMPs such as β-1,3-glucan (21), resulted in increased lung neutrophil recruitment and inflammatory cytokine gene expression in adiponectin-deficient mice, whereas Af293 aspiration had no significant difference (Figures 6B,E). In contrast with purified chitin, viable Af5517 conidia aspiration did not result in significantly increased eosinophil recruitment (Figure 6C). Initially, this appears to contradict our previous results (22). However, in the previous study, swollen and fixed Af5517 conidia were reported with significantly increased eosinophil recruitment after a single aspiration, whereas type 2 immune responses and eosinophil recruitment were markedly elevated after multiple aspirations of viable conidia. The differences in airway eosinophil recruitment after a single aspiration might be due to differences in temporal exposure of fungal PAMPs and concomitant immune recognition of viable vs. swollen/fixed conidia in both models. Furthermore, it is certain that Af293 and Af5517 exhibit phenotypic differences beyond cell wall composition, and thus differences between the strains can only be correlated with immune responses. Despite these caveats, we believe our data with particulate chitin and fungal conidia are consistent with broad targets for a regulatory action of adiponectin, especially in response to the combined stimulation of multiple PAMPs presented by germinating *A. fumigatus* conidia. Our future studies will examine the effects of adiponectin and roles of adiponectin receptors on the modulation of activation and inflammatory cytokine secretion in multiple cells types in response to co-recognition of multiple fungal PAMPs.

The global obesity epidemic and the association of obesity with a heightened inflammatory state has ignited widespread interest in the mechanisms of systemic and cellular immunometabolism (31). Many recent studies have focused on the effect of immune pathways in the regulation of adipose tissue and systemic metabolic homeostasis (31, 32). However, this relationship is reciprocal, as adipose tissue cytokines/hormones that were initially characterized as regulators of systemic metabolism, including adiponectin, are also known to play roles in immune regulation (33). Interestingly, we observed that chitin aspiration decreased serum levels of adiponectin and adipose tissue eosinophils (Figure 2D and unpublished data), suggesting that lung recognition of chitin affects metabolic and immune regulation at distal sites. Interestingly, a recent study reported that obese or adiponectin-deficient mice displayed decreased clearance of *Listeria monocytogenes* due to inhibition of chronic bone marrow inflammation (19). Furthermore, our results from a parallel study suggest the adiponectin dampens detrimental inflammation in invasive pulmonary aspergillosis (Amarsaikhan et al., submitted). Detrimental inflammation is also an important factor in allergic bronchopulmonary aspergillosis (ABPA), where lung airway persistence/colonization in susceptible individuals leads to inflammatory pathology mediated in part by eosinophils (34). However, the role of adiponectin in protection from ABPA remains unknown. In future studies, we aim to further explore the novel role for this pathway in dampening inflammation and improving survival in models of fungal inhalation, airway colonization, and invasive infection.

## Materials and Methods

### Mouse Strains

BALB/c, C57BL/6, and adiponectin-deficient (*adipoq*-/-) mice (B6 background) were received from Jackson Laboratory. SPAM transgenic mice with constitutive expression of acidic mammalian chitinase (AMCase) under the lung Clara cell-specific promoter *cc10* were provided by Dr. Richard Locksley [University of California, San Francisco (20)].

### Growth and Handling of Fungi

The clinical isolate Af293 was previously obtained from the Fungal Genetics Stock Center. The *A. fumigatus* isolate Af5517 was obtained from the United States Agriculture Research Service. Fungi were grown on Malt Extract Agar plates at 22°C and conidia suspensions were collected and aseptically prepared as described (21, 22).

### Mouse Aspiration of Chitin or Fungal Conidia

Custom-sized purified chitin particles (<70 μm) were obtained from New England Biolabs and prepared as previously described (20). Purified chitin particle suspensions were delivered by involuntary aspiration of 50 μl solution to isoflurane-anesthetized mice. For particles, 100 particles/μl were aspirated daily for 2 days and mice were sacrificed 24 h after final challenge to assess innate immune responses. For the adiponectin co-aspirations with chitin particles, recombinant adiponectin (Sino Biologicals) was reconstituted to the optimal concentration of 0.25 μg/Kg (x2 for a total of 0.5 μg/Kg) in Phosphate Buffered Saline (PBS) and aspirated into the airway alone or in combination with chitin particles. For repeated aspiration of conidia, 2 × 10^6^ conidia were aspirated and mice were sacrificed 72 h after the final challenge to assess inflammation and T cell-mediated responses as previously described (22). For single aspiration of conidia, 5 × 10^6^ Af293 or Af5517 conidia were aspirated and mice were harvested 48 h later to assess both neutrophil and eosinophil recruitment as well as inflammatory cytokine transcription (22, 35).

### Sample Collection and Processing

For transcription quantification, mouse lungs were harvested, flash frozen and used for total RNA extraction and analysis as previously described (22). Primers for qRT-PCR were obtained from SABiosciences. Serum was separated from blood collected by cardiac puncture and serum adiponectin levels were measured by ELISA according to manufacturer‘s instructions (R&D Systems). All flow cytometry reagents were obtained by BD biosciences or eBioscience, with the following exception: Rabbit mAb for mouse AdipoR1 was used along with IgG1 isotype control for primary stain followed by Goat anti-rabbit IgG Dylight 488 secondary antibody stain (both from Abcam). Populations of cells were evaluated by flow cytometric analysis on a Guava EasyCyte 8HT bench top flow cytometer (EMD Millipore) as previously described (22). For color compensation, mouse splenocytes were left unstained or stained with single color controls of rat anti-mouse CD4 antibodies.

### *In vitro* Eosinophil Culturing From Bone Marrow

Bone marrow derived eosinophil cultures were generated with bone marrow cells isolated from femurs of BALB/c mice, followed by incubation with recombinant SCF, FLT3L, and IL-5 as described by Dyer et al. (36). Before use in experiments, differentiated eosinophils were confirmed using cytospin followed by with histology staining (DiffQuick) and flow cytometry for Siglec-F expression. Mature eosinophils after Day 12 were enumerated and used for chemotaxis assay and total RNA isolation. For chemotaxis assay, eosinophils were incubated with 5 μg/ml recombinant adiponectin (R&D) for 60 min. Migration was measured by counting cells on hemocytometer after cells were incubated on transwell (5 μm pore size, Costar) with bottom chamber media containing 100 ng/ml eotaxin. Positive control with eotaxin-1 (CCL11) in both chambers and negative control with no chemokine was included. For gene expression of *adipoR1* and *adipoR2* total RNA was isolated from cells treated with and without adiponectin for 4 h. Total RNA isolation was performed with Trizol (Ambion) method combined with RNAeasy mini purification columns according to manufacturer‘s protocol (Qiagen).

### Lung Epithelial Cell Culture

The human lung epithelial cell line A549 was obtained from ATCC and cultured according to supplier‘s protocol in F-12K media with 10% FBS at 37°C with 5% CO_2_. For experiments, 1 × 10^6^ cells/ml were seeded overnight in no serum growth media to arrest growth. Next day, media was replaced with regular serum media with or without 100 ng/ml recombinant human TNFα (R&D Systems) [to induce adiponectin expression as previously described (27)] alone or co-incubated with purified chitin particles (5,000 particles/ml) or 1 × 10^7^ Af293 or Af5517 conidia for 6 h (MOI 10). Post incubation, media was removed and cells were lysed in 1 ml Trizol. Total RNA was isolated with Qiagen RNAeasy columns, cDNA synthesized and used for qRT-PCR for gene expression analysis. Secreted cytokines from cell free culture supernatants were quantified using ELISA kits (Peprotech) according to manufacturer‘s protocols.

### Data Analysis Methods

Analysis of mouse flow cytometric data was performed with FlowJo software, version 10 (Becton-Dickinson). Prism 6 software was used for generation of graphs and figures and for statistical analyses (GraphPad). Unpaired *t*-tests were used to measure statistical significance when two groups were directly compared, and one or two-way analysis of variance (ANOVA) tests were used for comparison of three or more groups, followed by Tukey's or Sidak's post-tests for multiple comparisons, respectively. Differences between experimental groups that resulted in a *p* < 0.05 were considered significant.

## Ethics Statement

This study was carried out in accordance with the recommendations of the PHS Policy on Humane Care and Use of Laboratory Animals. The protocol was approved by the Indiana State University Animal Care and Use Committee, the host campus of IUSM-Terre Haute.

## Author Contributions

ST conceived the project and wrote the paper. ST and NA designed the experiments. NA, DS, AW, ES, AT, and HG performed the experiments.

### Conflict of Interest Statement

The authors declare that the research was conducted in the absence of any commercial or financial relationships that could be construed as a potential conflict of interest.

## Supplementary Material

Figure S1 Lung leukocyte recruitment, adiponectin, and leptin transcription 24 h after a single aspiration with 3 mg curdlan (in 50 μl saline). **(A)** Total cells, **(B)** neutrophils, **(C)** eosinophils, **(D)** adiponectin (adipoq) expression by qRT-PCR (Saline control baseline). **(E)** Leptin expression in saline, curdlan, and chitin-aspirated mice. **(D,E)** *N* = 4/group. ^***^*p* < 0.001. ^****^*p* < 0.0001.

Figure S2 Adiponectin, AdipoR1, and selected cytokine expression in human lung epithelial A549 cells in response to purified chitin or *A. fumigatus* conidia. A549 cells were incubated for 6 h with or without human recombinant TNF and/or purified chitin, or swollen/fixed *A. fumigatus* conidia from Af293 or Af5517 isolates as described in Materials and Methods. **(A–C)** Messenger RNA quantification by qRT-PCR of the indicated genes in cell lysates. **(D)** Quantification of protein levels of indicated cytokines from cell supernatants by ELISA. Data are a summary of two experiments with *N* = 6/group. ^*^*p* < 0.05. ^**^*p* < 0.01. ^****^*p* < 0.0001.
