# Supplementary figures and images for: Reciprocal Inhibition of Adiponectin and Innate Lung Immune Responses to Chitin and Aspergillus fumigatus
Source: Front Immunol. 2019 May 10;10:1057. doi: 10.3389/fimmu.2019.01057 (PMC6524459; doi:10.3389/fimmu.2019.01057)

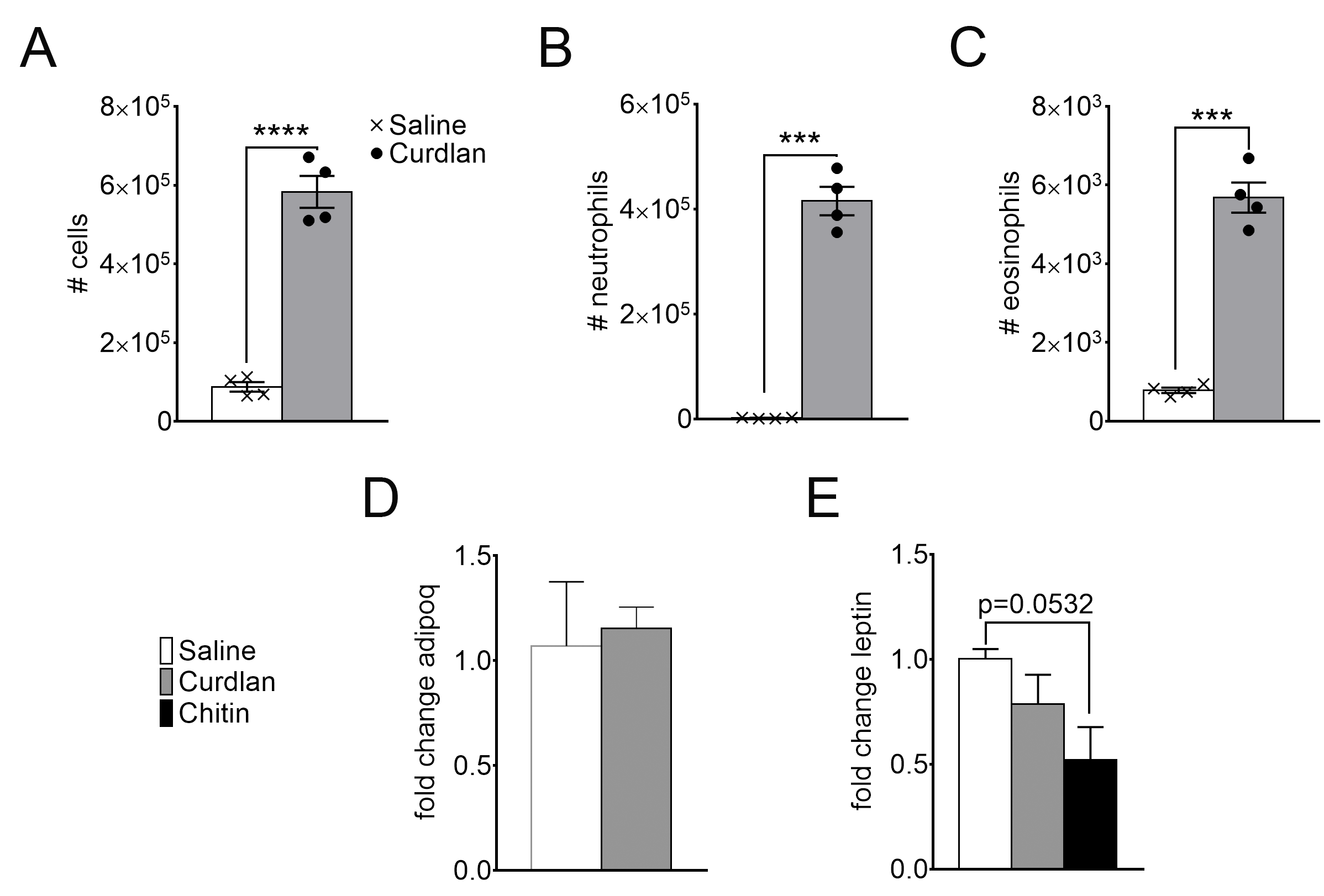

Supplement: Figure S1 — Lung leukocyte recruitment, adiponectin, and leptin transcription 24 h after a single aspiration with 3 mg curdlan (in 50 μl saline). (A) Total cells, (B) neutrophils, (C) eosinophils, (D) adiponectin (adipoq) expression by qRT-PCR (Saline control baseline). (E) Leptin expression in saline, curdlan, and chitin-aspirated mice. (D,E) N = 4/group. ***p < 0.001. ****p < 0.0001. [file Image_1.TIF]

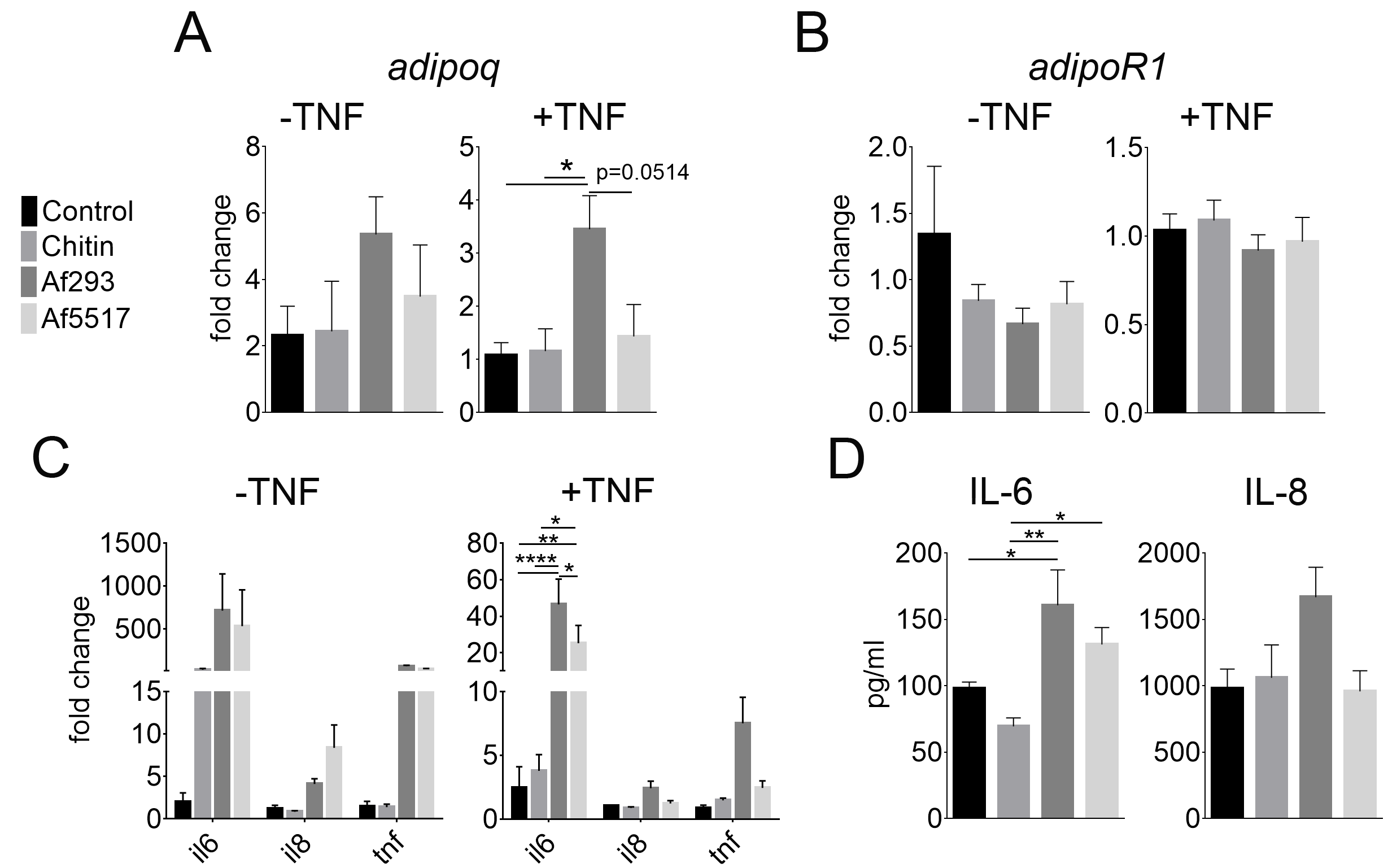

Supplement: Figure S2 — Adiponectin, AdipoR1, and selected cytokine expression in human lung epithelial A549 cells in response to purified chitin or A. fumigatus conidia. A549 cells were incubated for 6 h with or without human recombinant TNF and/or purified chitin, or swollen/fixed A. fumigatus conidia from Af293 or Af5517 isolates as described in Materials and Methods. (A–C) Messenger RNA quantification by qRT-PCR of the indicated genes in cell lysates. (D) Quantification of protein levels of indicated cytokines from cell supernatants by ELISA. Data are a summary of two experiments with N = 6/group. *p < 0.05. **p < 0.01. ****p < 0.0001. [file Image_2.TIF]
